# Surgery Is the Last Resort for Huge Scrotal Lymphedema: A Series of Challenging Cases

**DOI:** 10.1055/s-0042-1757572

**Published:** 2023-02-10

**Authors:** Yasser M. ElKiran, Amr M. Elshafei, Mohamed S. Abdelgawad, Mohammed F. Kamel, Hesham A. Sharaf-Eldeen, Mohammed A. Abdelmaksoud

**Affiliations:** 1 Department of Vascular and Endovascular Surgery, Faculty of Medicine, Mansoura University, Mansoura, Egypt

**Keywords:** lymphedema, genitalia, scrotal swelling

## Abstract

We aim to provide our surgical techniques, and outcomes of functional scrotal reduction procedures with complete preservation of the genitourinary original anatomy in a simple way without using complicated skin grafting or skin advancement flaps in Patients with huge and long-standing scrotal lymphedema 18 patients ages ranged from 14-65 with a median of 30 years. Functional scrotal and penoscrotal reduction was attained in all cases, without distortion of the genitourinary anatomy and without the need for advancement, rotational or free flaps, maximal scrotal diameter was reduced from median of 61[48-92] cms to a median of 25[21–29] cms ( *P* <0.0001) and remained almost unchanged at the end of the follow up period 26[22-34] cms ( *P* <0.0001). Sexual performance and voiding capacity were improved in all patients, testicular vascularity was unaffected and the Glasgow Benefit Inventory (GBI) for the quality of life showed marked enhancement in the total 55.5[50–72], general 55.5[50–72], social 100[50–100] and physical 16.6[16–33] points subscales. According to our experience, surgery remains the gold standard treatment for management of huge scrotal lymphedema, successful preservation of the genitourinary functions can be attained despite the size in most cases with excellent cosmoses.

## Introduction

Scrotal lymphedema, a rare entity of lymphedema, is defined as abnormal accumulation of a protein-rich fluid in the soft-tissue region of the scrotal/penoscrotal territory coupled with gross deformation of the genitalia 1 because of a derangement of the lymphatic drainage. Lymphedema is classified as primary (idiopathic) or secondary according to its etiology. Primary lymphedema is caused by an intrinsic defect of the lymphatic vessels, while secondary lymphedema may occur after surgery, radiation, tumors, and infections. 2

Scrotal lymphedema produces mobility and voiding limitations, fatigue, pain, and recurrent subcutaneous infections because of the difficulty of self-hygiene. It also causes sexual limitations and social isolation and impairs quality of life. 3 4 5 Therapy is usually initiated by conservative measures, for example, the complex decongestive physiotherapy (CDP) first described by Földi, including skin hygiene, manual lymph drainage, compression bandages, and therapeutic exercises. 6 7 However, many patients experience either unsatisfactory results or recurrence even after minor size reduction. 8 9

Reported methods of surgical reconstruction involve either lymphangioplasty or complete excision of lymphedematous tissue with local tissue reconstruction in case healthy skin is available. Other methods of reconstruction will include essentially advancement flaps, rotational flaps, and split- or even full-thickness free flaps, whose results in such area exposed to many commensals and uncontrollable hygiene were questionable. 3 10

It has been established that surgical reduction of scrotal lymphedema achieves a significant improvement in quality of life provided the genitourinary anatomy and physiology remains unaltered. 11

In this series of challenging cases, we aimed to share our experience with functional scrotal reduction without using advancement, rotational, or free flaps while preserving the structure and function of the penoscrotal region, and demonstrating its impact on the patient wellness.

## Idea

In this retrospective study, we present our experience with huge scrotal lymphedema cases that were treated with surgical reduction. All patients consented verbally and formally for photographing their pre- and postoperative conditions and their approval was submitted in the Institutional Research Board of the Faculty of Medicine, Mansoura University, under approval number R/18.12.373.

### Operative Technique

Our surgical technique was previously described in our published case reports of huge scrotal lymphedema patients who underwent successful reduction scrotoplasty despite having a huge-sized scrotum; these cases were included in our case series. 12 13 It depended principally on the presence of surrounding healthy scrotal skin that allowed sufficient flaps opposition and suffice wound healing without involving complex regional rotational, advancement, or free flaps that rendered the healing questionable. The penile urethra was secured by a Foley catheter prior to the commencement of the procedure and remained there postoperatively to facilitate voiding for 3 to 10 days postoperatively.

**Fig. 1:**
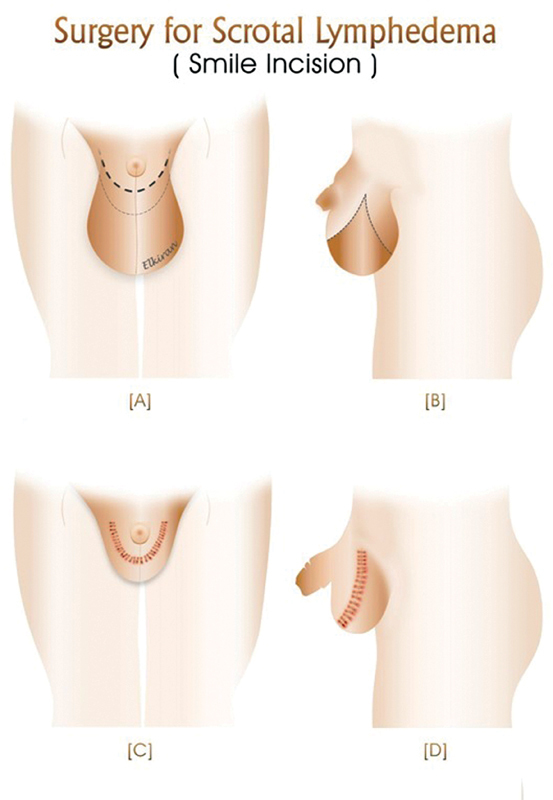
Our classic smile incision and creation of the upper and lower flaps from anterior and lateral views ( **A, B** ), and postoperative images after closure of the skin ( **C, D** ).

Our classic “smilelike” incision ( Fig. 1 ; Supplementary Video S1 , Debulking technique for huge scrotal lymphedema management, available in the online version only) extended typically short below the external inguinal ring from one side to the other, curving 1 inch below the penoscrotal sulcus until it reached the other side. The lower incision extended between the exact two previous points but was taken down and posteriorly to involve much of the scrotal skin to be excised and underlying lymphedematous tissue.

**Fig. 2:**
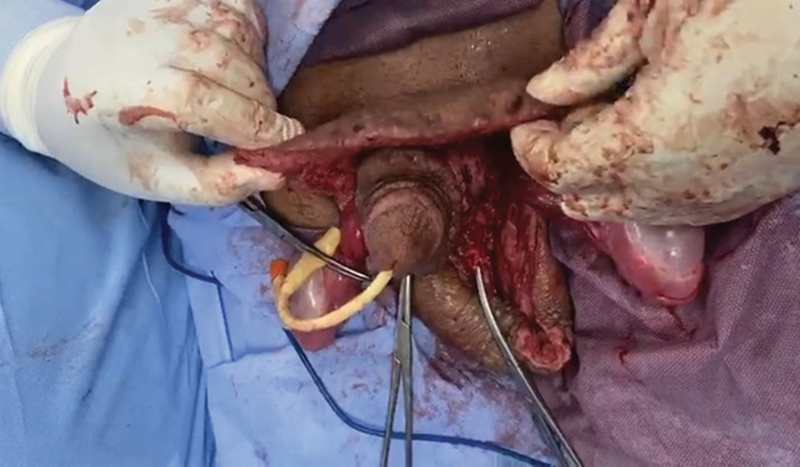
Alternate incision in case of excess lymphedematous suprapubic tissue.

Modification to such incision was undertaken in cases that exhibited significant lymphedematous tissue in the suprapubic region, which obliterates the penopubic junction and buries the penis, displacing the perpetual opening downward. In such cases we designed the anterior flap from the over-hanged skin, excess subcutaneous tissue removed, the dissected penis with small scrotal skin cuff was transposed proximally to its anatomical site either brought out through a button hole or after dividing the anterior flap in the mid-line converting it to a butterfly, the penis with its cuff was repositioned between the two wings ( Fig. 2 ).

**Fig. 3:**
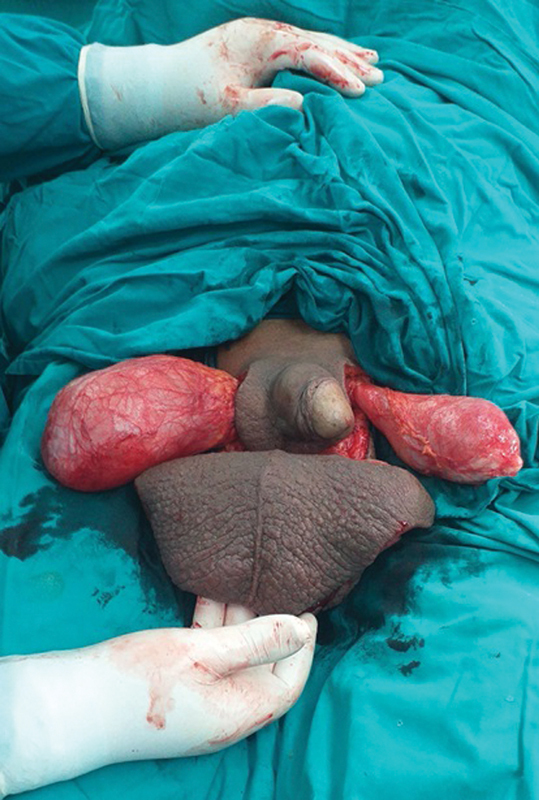
Dissection and isolation of the testicles and cord in a case with concomitant bilateral hydrocele.

**Fig. 4:**
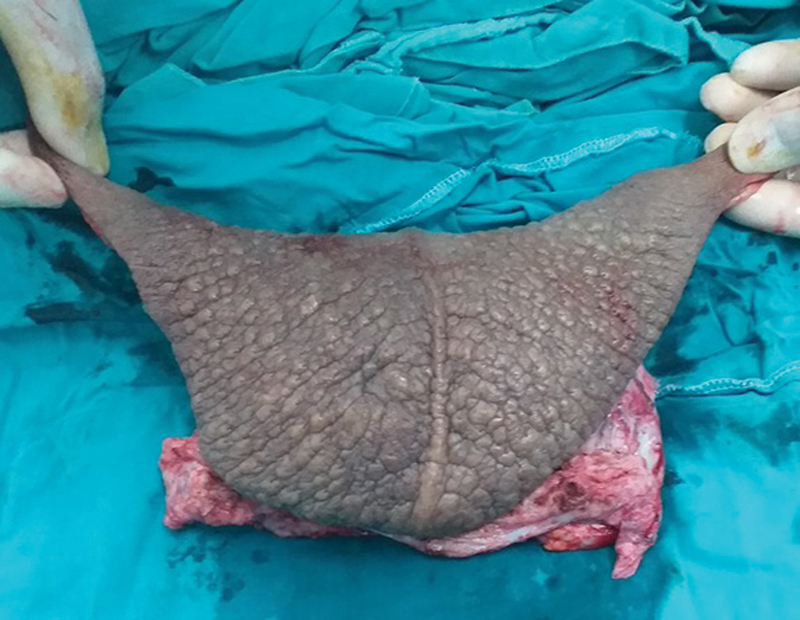
En bloc excision of the anterior scrotal skin together with the lymphedematous tissue after smilelike incision.

After creation and dissection of the flaps, the testicles and cord components were carefully dissected ( Fig. 3 ) and secured by vessel loops until the time of flap closure. The lymphedematous tissue was excised principally from the anterior scrotal surface area and was removed en bloc ( Fig. 4 ).

The tunica vaginalis was everted routinely. However, in cases with concomitant hydrocele, it was excised subtotally, thus promoting lymphatic drainage of the scrotal skin via testicular lymphatics into the nondiseased para-aortic lymph nodes. 14

**Fig. 5:**
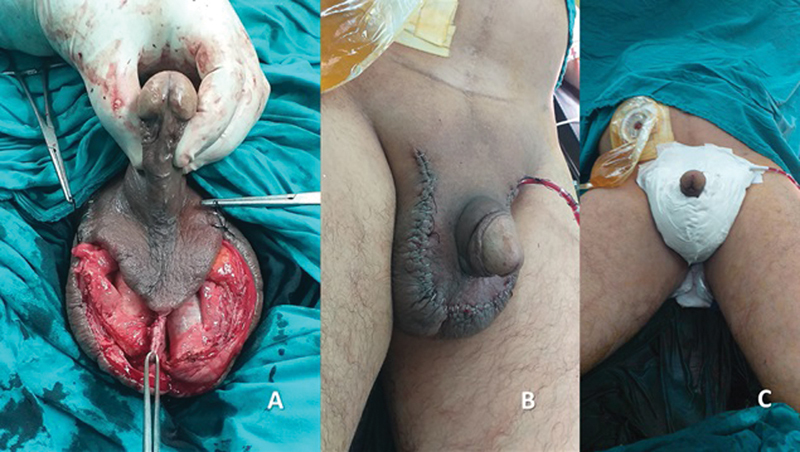
Thinning of the scrotal septum and commencement of flap opposition ( **A** ), then closing the skin with staples ( **B** ), and packing both sides of the scrotum ( **C** ).

The scrotal septum was preserved but thinned out. Then, the flaps were reassessed and trimmed to provide the least possible size after completion of scrotoplasty. V-excision was performed from the lower flap if required. Closure was performed by means of a skin stapler afterward ( Fig. 5 ), and draping tapes with scrotal elevation were used to minimize edema.

**Fig. 6:**
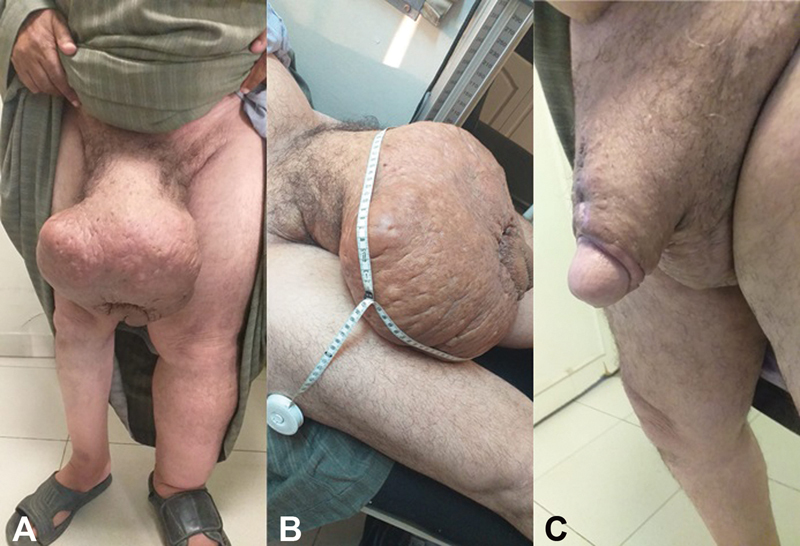
A 65-year-old man presented with huge scrotal and penile lymphedema, 20 years ago after two debulking surgeries, and concomitant left lower limb lymphedema ( **A** ). The maximum diameter of the swelling was 92 cm ( **B** ). 18 months postoperative follow-up image ( **C** ).

In the case of coexisting penile lymphedema, the stretched healthy preputial skin was used to cover the penile shaft; in case of shortage of preputial skin, we covered the rest of penile shaft with local skin flap ( Fig. 6 ).

### Follow-up

During the follow-up period, we encouraged our patients to commit to a self-management disciplinary program to maintain best outcomes. Our protocol comprised four components: (1) improving self-hygiene and thorough skin care; (2) self-performed manual lymphatic drainage; (3) deep breathing exercises (it had a lymphokinetic action); and (4) self-performed manual intermittent scrotal compression. In our novel method of scrotal compression, the patient was instructed to lie down on a longitudinally folded linen, half of it underneath his back and buttocks and the rest of it passing under the patient's lap underneath the scrotum. The patient himself pulls the folded linen cranially, compressing the scrotum for 1 to 2 minutes, and then releases it, repeating for 10 times.

Patients had weekly follow-up visits during the first month, then every 3 months until the first year, and then annually until 3 years of follow-up, where serial circumferential measurements of the scrotum were taken ( Supplementary Appendix 1 , available in the online version only). Assessment of voiding capacity and sexual functionality (in sexually active individuals) was done in all patients in all visits through some questions, which the patients answer with yes/no ( Supplementary Appendix 2 , available in the online version only). Satisfaction after the operation was assessed using the Glasgow Benefit Inventory (GBI), which was filled by the patients themselves after the end of follow-up period, as adopted by some authors previously. 15

The results were assessed by the GBI, an established questionnaire that assessed how a procedure had altered the quality of life of the patient. The GBI not only allowed the calculation of “total” quality of life, as influenced by the intervention, but also permitted a breakdown of the results into “general subscale,” “social support subscale,” and “physical health subscale.”

The score was calculated as follows: the sum of the responses is first divided by the number of questions in the respective subscale to get an average response score. From the average response score, three was subtracted and the result is multiplied by 50. This gave a score between −100 and 100, with −100 being the worst possible change, 0 no change, and 100 the best possible change. 15

### Statistical Analysis

Numerical values were described as mean and standard deviation when normally distributed and as median and interquartile range if abnormally distributed. Repeated measures for the entire sample pretreatment, after CDP, after surgery, and in each follow-up visit were recorded and analyzed using SPSS (version 22.0) according to the appropriate test.

### Results

From January 2014 until July 2018, we performed 18 successful scrotal and penoscrotal functional reduction surgeries for cases condemned to be hopeless because of the apparently huge scrotal diameter or the long-standing condition. The ages of our patients ranged from 14 to 65 years, with a median of 30 years. The maximum scrotal diameter ranged from 48 to 92 cm with a median of 61 cm preoperatively at the time of presentation.

We had seven cases associated with lower limb lymphedema; two of these were diagnosed as lymphedema congenita, while five were considered as lymphedema praecox. One patient had trisomy 21 and was on corticosteroid therapy, one patient had giant cell nevus syndrome, one patient had Rosai–Dorfman disease, and one patient had secondary lymphedema that developed postradical cystectomy 6 years before our intervention. Three patients had concomitant testicular hydrocele and two patients had penile lymphedema as well. Two patients had recurrent scrotal swelling after previous surgical excision.

Successful reduction from a median size of 61 cm (range, 48–92 cm) to 25 cm (range, 21–29 cm) ( *p* < 0.0001) with total preservation of the genitourinary anatomy was attained immediately postoperatively. During the follow-up period, which stretched until 72 months postintervention, the size varied between slight insignificant decreases and increases ( Table 1 ). Such significant reduction in size remained until the end of the follow-up period (median, 26; range: 22–34 cm) ( *p* < 0.0001 between the maximal scrotal diameter preoperatively and 3 years postoperative). In two patients, the penile skin was grossly damaged, so we used the stretched preputial skin to cover the penis in one case and a local flap constructed from the hairless stretched penopubic junction for the second case. The median operative time was 160 minutes (range, 120–360), and the median total estimated blood loss was 200 mL (range, 120–750).

**Table 1:** Median, minimum, and maximum of the maximal scrotal preoperatively and throughout the follow-up period

	Diameter			
Time	Median (cm)	Minimum	Maximum	*p* -Value
Preoperative	61	48	92	<0.0001
Postoperative	25	21	29	
3 mo	29	25	39	
6 mo	28	24	35	
9 mo	28	24	32	
24 mo	28	23	32	
36 mo	26	22	34	

Two patients developed wound dehiscence, which caught minor infection in one patient and was clean in the other. Both were treated with systemic antibiotics besides local wound care and repeated dressing until healing by secondary intention was achieved. Two other patients developed minor flap edge necrosis, which also was handled conservatively under antibiotic coverage until secondary closure was safe.

All patients showed absolutely no problems with micturition. Among those who were sexually active, all except four had significantly more satisfactory intercourse postoperatively. Therefore, answers for posed questions in the box ( Supplementary Appendix 2 , available in the online version only) were positive for the first 3 questions for 14 patients. All patients experienced no chronic postoperative pain after the surgery (replied negatively for the fourth question in the box) ( Supplementary Appendix 2 , available in the online version only). Scrotal duplex showed testicular vascularity was unaffected in all cases. The previous factors were assessed in each postoperative visit by answering the questions in box ( Supplementary Appendix 2 , available in the online version only). Scrotal duplex was performed during the first postoperative visit only.

**Fig. 7:**
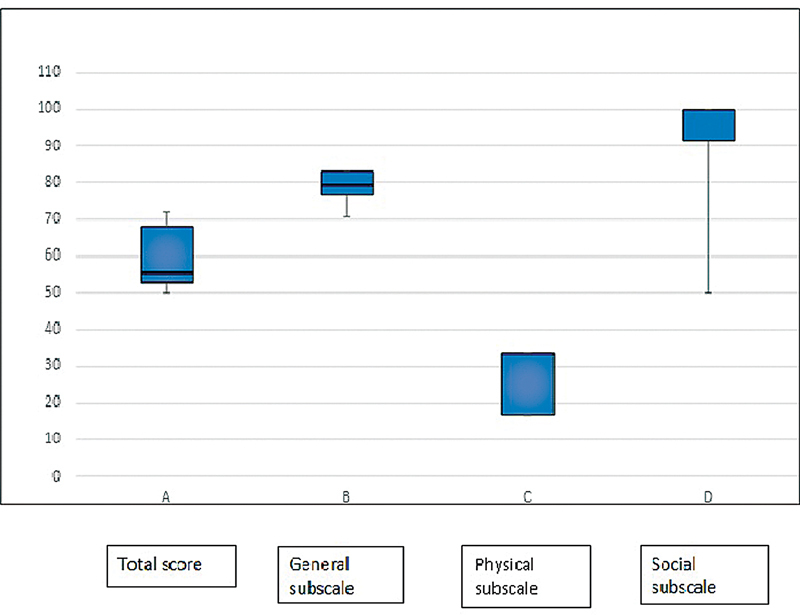
Box and whisker plot for the total, general, physical, and social subscales of the GBI.

The GBI was used at the end of the follow-up period to assess the true benefit of functional scrotal reduction and its impact on the physical wellbeing and the social support the patient receives after the operation. The analysis of the GBI questionnaire was performed by members of our team other than those involved in the scrotal reduction operation. The total score was 55.5 (range, 50–72). It showed that the physical wellbeing increased by a median of 16.6 points (range, 16–33 points), the social support the patients received increased by a median of 100 points (range 50–100 points), and the general wellbeing subscale also showed an increase by a median of 79 (range, 70–83 points) ( Fig. 7 ).

## Discussion

The problem of lymphedema affecting the region of the scrotum and penis can lead to significant harm on the patients' personal hygiene, sexual capability, and desire to participate and interact with the surrounding environment. Also, faulty medical practice of treating such cases conservatively despite the absence of an obvious improvement can lead to significant despair that withholds the patient from seeking proper medical advice.

We presented 18 challenging cases of scrotal lymphedema that suffered from huge diameters of the scrotal sac (smallest was 48 cm). The fact that we pose certain conditions to define functional scrotal reduction surgery is derived from our caution to preserve the genitourinary functions of the penis and scrotum despite the size. We were never bailed out to orchidectomy or urinary diversion in any case no matter how deep the penis was buried or the genitalia were deformed.

The presence of minimal surface area of healthy scrotal skin was mandatory to reconstruct the scrotum without needing advancement, rotational, or distal split- or full-thickness skin flaps. 16 17 18 In our technique, we reconstructed the new scrotal sac mainly using the posterior flap. This posterior flap resembles the most healthy area in scrotal lymphedema (the uppermost stretched posterior skin by the hanged swelling with less edema). This condition was questionable in two cases (one patient with Rosai–Dorfman disease and one patient with giant cell nevus syndrome) because of the presence of unhealthy velvety scrotal skin that rendered healing doubtful; however, successful reconstruction was also attained according to our conditions.

The significant reduction in the size of the scrotum postintervention was maintained throughout the follow-up period that continued for 3 years. Slight alteration in the median size in between was insignificant and was attributed to development of postoperative edema, and its resolution over time until the follow-up period was considered complete. Our protocol, which was based on self-management discipline, gave satisfactory outcomes maintained throughout the follow-up period, avoiding slow gradual recurrence. We did not subscribe compression hosiery or garments (shorts, boxers, pouches, support, or pads) to any of our patients, thus avoiding the costly, painfully tight garments with its bad hygienic effect, thus allowing our patients to resume a normal life.

The improvement of the physical, sexual, and voiding capacity was investigated in each postoperative visit by asking the patient to respond to four questions (whether he can void freely/whether his sexual performance was improved/whether he can walk freely/whether he suffered any chronic postoperative pain) and the reply was positive for all questions for all patients for the first three questions except for four patients who were sexually inactive. Patients also denied the presence of any chronic regional postoperative pain after the surgery (question 4). We went further to investigate the overall benefit from scrotal reduction surgery using the GBI, adopted by some authors in previous similar work. 5 This also conveyed excellent results on the physical wellbeing, the social support received, and overall performance. The social support the patients received increased by 100 points (range, 50–100). Many of them were inclined not to participate in social activities and were less confident in job opportunities. This has changed radically, for instance.

The physical wellbeing also improved by a median of 16.6 points (range, 16–33 points), perhaps because the three questions of physical wellbeing targeted the intake of drugs, family doctor visits, and frequency of cold infections. This is why, the impact was not evident as in the social support sector, which was more detailed. The general wellbeing sensation increased as well by a median of 79 points (range, 70–83 points), surpassing similar studies using GBI in assessment.

The technique adopted by our lymphedema team was developed over a long period of time and thorough experience with a huge number of lymphedema cases either in the extremities or in the scrotum. For example, we adopted the smilelike incision and its butterfly modification to create flaps that would oppose without the risk of flap ischemia or necrosis such that minimal postoperative care would be required postintervention. The appropriate repositioning of the penile shaft in the exact or near-exact anatomical position is crucial for hygienic voiding. Therefore, the modified butterfly incision was used in cases where penile shaft would be extremely downward displaced after closure of the flaps. Also, V-incisions to excise excess redundancy of posterior flap were used according to the operator discretion whenever needed. The appropriate repositioning of the penis and its stretching from the surrounding tissue would ensure better voiding and intercourse functions. According to our experience, surgery remains the gold standard treatment for the management of huge scrotal lymphedema. Successful preservation of the genitourinary functions can be attained despite the size in most cases with excellent cosmesis. Our study is limited by its retrospective nature and the relatively few numbers of cases, which might have distorted the statistics. However, since we were dealing with a relatively rare entity and discussing only cases whose maximal scrotal diameter is approximately 48 cm in the mildest case, such number is acceptable.
