# Supplementary material for: Surgery Is the Last Resort for Huge Scrotal Lymphedema: A Series of Challenging Cases
Source: Arch Plast Surg. 2023 Feb 10;50(2):182–7. doi: 10.1055/s-0042-1757572 (PMC10049820; doi:10.1055/s-0042-1757572)
Supplement: Supplementary file 1 — Supplementary Material [file 10-1055-s-0042-1757572-s22jan0000oa.pdf]

## Supplementary Appendix 1

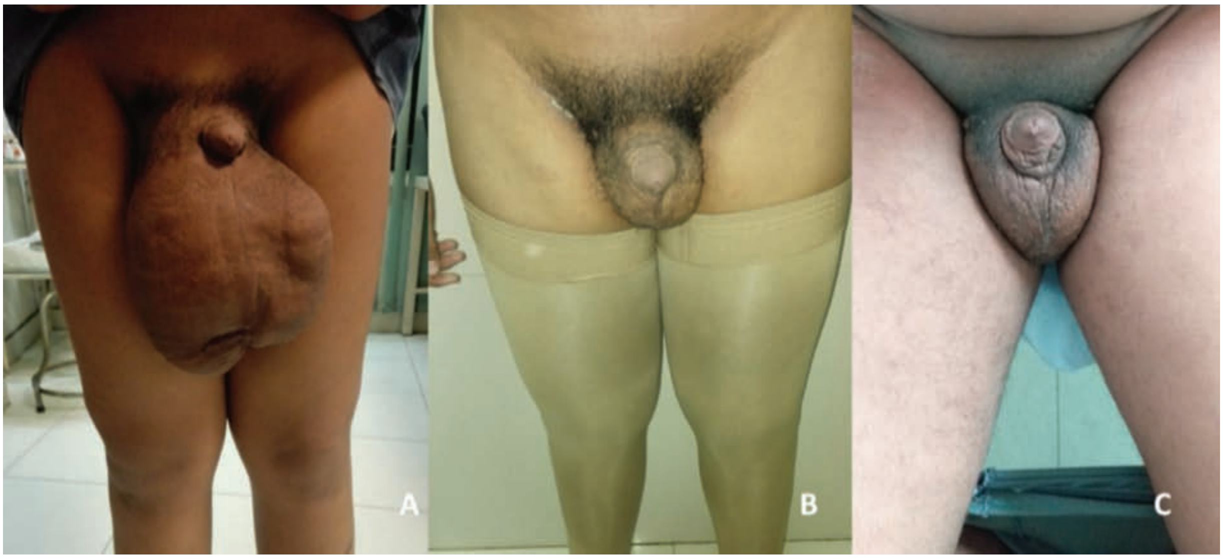


**Fig. 8** Case 1: A 14-year-old male patient with a maximal scrotal diameter of 68 cm, which was associated with lymphedema (congenital) of both the lower limbs on CDT, preoperatively (**A**) and one year postreduction (29 cm) (**B**) and 3 years postreduction (26 cm) (**C**).

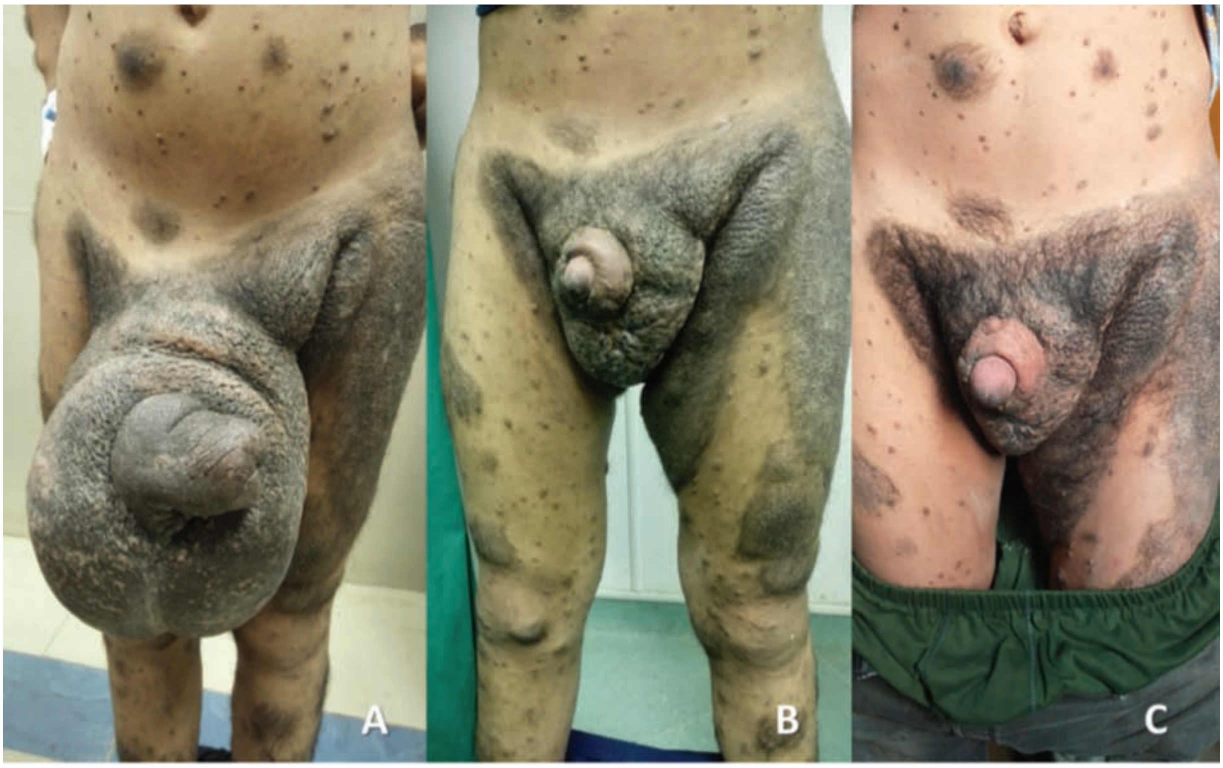


**Fig. 9** Case 2: A 15-year-old male patient with giant cell nevus syndrome showing scrotal lymphedema dating since birth with maximal scrotal diameter of 75 cm preoperatively (**A**). One year postreduction (26 cm) (**B**) and 3 years postreduction (25 cm) (**C**).

 © 2023. The Korean Society of Plastic and Reconstructive Surgeons. All rights reserved.

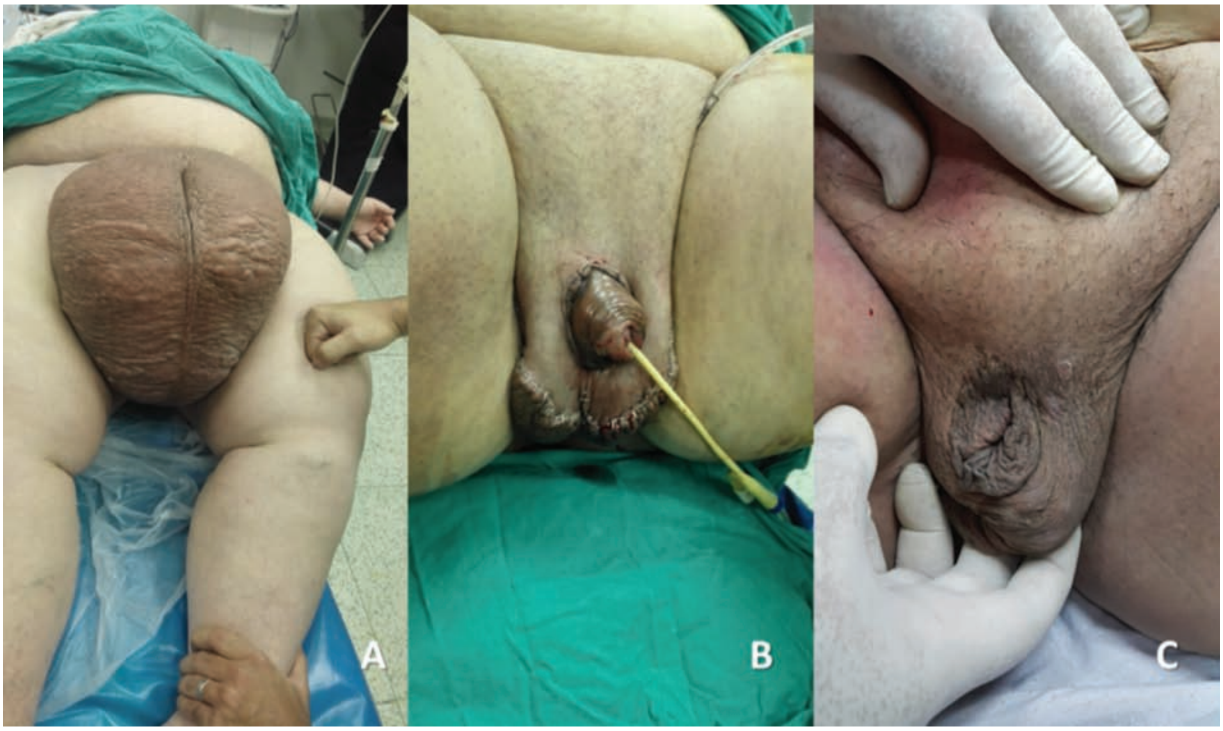


**Fig. 10** Case 3: A case of huge scrotal lymphedema in a morbidly obese patient with trisomy 21 measuring 82 cm in the maximum scrotal diameter preoperatively (**A**). Immediately postoperative image (26 cm) with buttonhole modification to anterior flap (**B**). After 1 year (**C**).

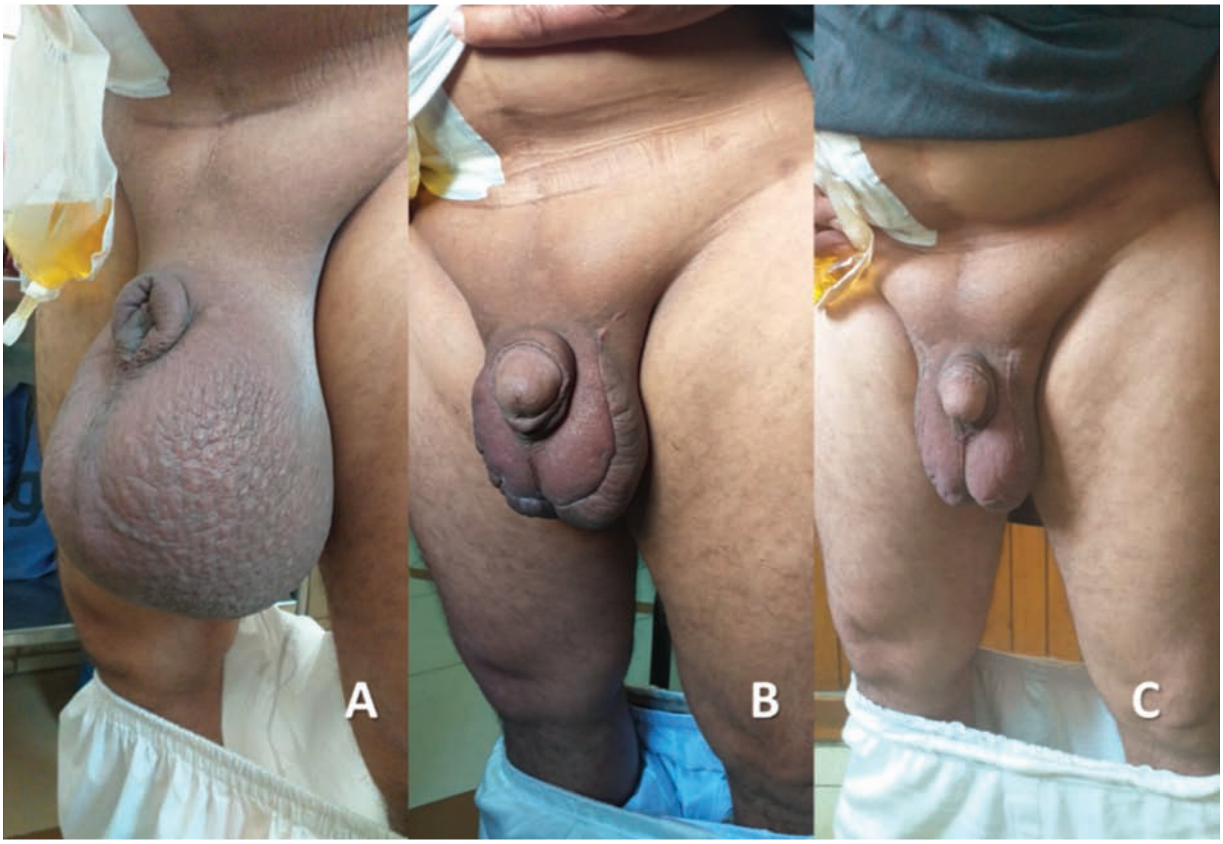


**Fig. 11** Case 4: A 53-year-old man with a past history of radical cystectomy and urinary diversion 14 years ago presented with huge scrotal swelling 2 years ago and recurrent erysipelas (four attacks per year) (**A**). Early postoperative images after scrotal debulking (**B**). Late postoperative image (**C**).

 © 2023. The Korean Society of Plastic and Reconstructive Surgeons. All rights reserved.

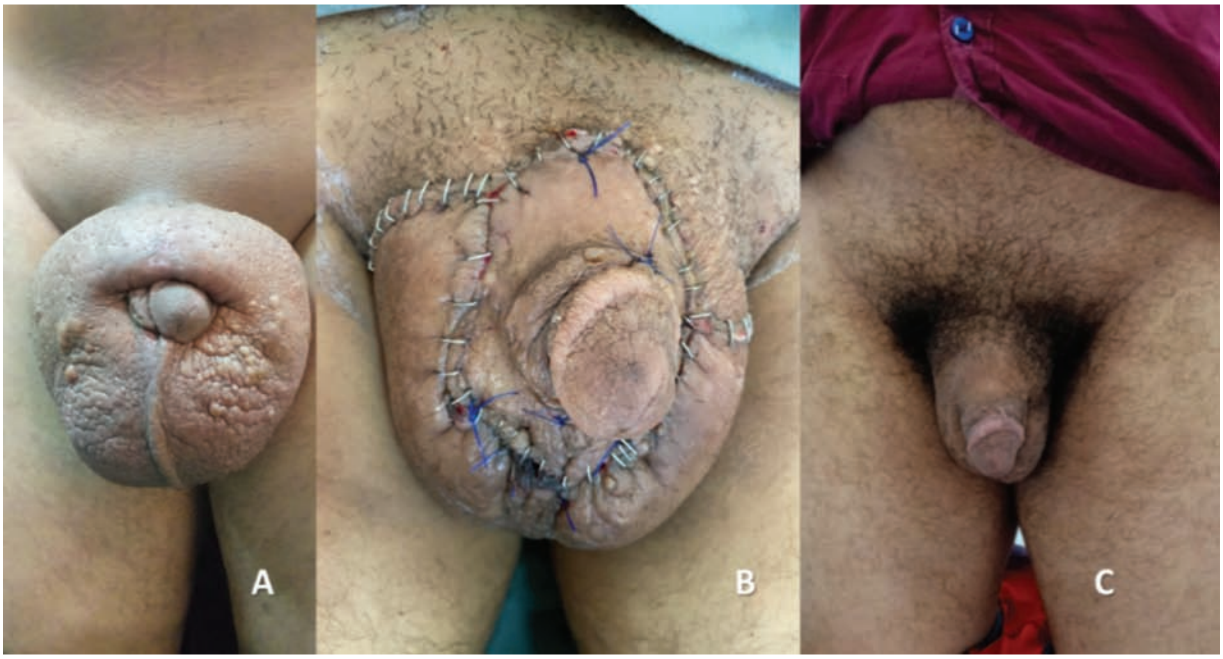


**Fig. 12** Case 5: An 18-year-old man presented with scrotal swelling 5 years ago with no obvious cause (lymphedema praecox) (**A**). Butterfly modification for anterior flap (early postoperative image) (**B**). Late postoperative image (**C**).

 © 2023. The Korean Society of Plastic and Reconstructive Surgeons. All rights reserved.

Summary table for the case series

| Case no. | Age (y) | Duration | Cause and associated morbidities | Preoperative scrotal circumference (cm) | Scrotal circumference at the end of follow-up (cm) | Procedure | Operative time (min) | Blood loss (mL) |
|---|---|---|---|---|---|---|---|---|
| 1 | 18 | Since birth | Primary (congenital) | 59 | 26 | Scrotal debulking | 180 | 200 |
| 2 | 53 | 2 y | Secondary (radical cystectomy) | 63 | 29 | Scrotal debulking | 210 | 300 |
| 3 | 21 | 2 y | Bronchial asthma on corticosteroid therapy, trisomy 21, morbid obesity | 82 | 26 | Scrotal debulking | 160 | 150 |
| 4 | 15 | 12 y | Giant cell nevus syndrome | 75 | 26 | Scrotal debulking + penile reconstruction | 360 | 750 |
| 5 | 14 | 8 y | Primary (praecox), bilateral lower limb lymphedema | 68 | 29 | Scrotal debulking | 180 | 250 |
| 6 | 65 | 36 y | Primary (praecox), left lower limb lymphedema | 92 | 24 | Scrotal debulking+ penile reconstruction | 290 | 700 |
| 7 | 32 | 6 y | Morbid obesity, recurrent infections | 86 | 28 | Scrotal debulking | 120 | 400 |
| 8 | 45 | 4 y | Recurrent infections | 69 | 22 | Scrotal debulking | 155 | 250 |
| 9 | 44 | 16 y | Primary (praecox), left lower limb lymphedema | 57 | 22 | Scrotal debulking | 150 | 180 |
| 10 | 28 | Since birth | Primary (congenital), right lower limb lymphedema | 50 | 22 | Scrotal debulking | 150 | 200 |
| 11 | 40 | 12 y | Primary (praecox) | 62 | 26 | Scrotal debulking | 160 | 150 |
| 12 | 60 | 1 y | Thrombophlebitis of marked left varicocele | 78 | 23 | Scrotal debulking | 160 | 300 |
| 13 | 18 | 5 y | Primary (praecox), | 51 | 22 | Scrotal debulking | 220 | 120 |
| 14 | 19 | Since birth | Primary (congenital), bilateral lower limb lymphedema | 50 | 24 | Scrotal debulking | 140 | 150 |
| 15 | 24 | 7 y | Primary (praecox) | 60 | 34 | Scrotal debulking | 150 | 400 |
| 16 | 16 | 10 years | Primary (praecox), Bilateral lower limb lymphedema | 48 | 26 | Scrotal debulking | 220 | 120 |
| 17 | 39 | 12 years | Primary (praecox), left lower limb lymphedema | 52 | 28 | Scrotal debulking | 150 | 150 |
| 18 | 38 | 4 y | Rosai–Dorfman disease | 50 | 22 | Scrotal debulking | 180 | 150 |

 © 2023. The Korean Society of Plastic and Reconstructive Surgeons. All rights reserved.

**Supplementary Appendix 2** Questions posed during each follow-up visit.

| | |
|---|---|
| Can you void in standing position? | |
| Yes | No |
| Can you stand and walk freely? | |
| Yes | No |
| Has your sexual performance/has sexual intercourse become easier since the surgery? | |
| Yes | No |
| Is there any chronic pain after the surgery? | |
| Yes | No |
| Has the genital hygiene maintenance become easier after the surgery? | |
| Yes | No |

 © 2023. The Korean Society of Plastic and Reconstructive Surgeons. All rights reserved.
